# Intra-fraction motion of the prostate is not increased by patient couch shifts

**DOI:** 10.1186/s13014-016-0620-z

**Published:** 2016-03-22

**Authors:** Hendrik Ballhausen, Ute Ganswindt, Claus Belka, Minglun Li

**Affiliations:** University Hospital of LMU Munich, Department of Radiation Oncology, Marchioninistraße 15, 81377 Munich, Germany

**Keywords:** Radiotherapy, Prostate, Intra-fraction motion

## Abstract

**Background:**

During a fraction of external beam radiotherapy for prostate cancer, a mismatch between target volume and dose coverage may accumulate over time due to intra-fraction motion. One way to remove the residual error is to perform a couch shift in opposite direction. In principle, such couch shifts could cause secondary displacements of the patient and prostate. Hence it is interesting to investigate if couch shifts might amplify intra-fraction motion.

**Findings:**

Intra-fraction motion of the prostate and patient couch position were simultaneously recorded during 359 fractions in 15 patients. During this time, a total of 22 couch shifts of up to 31.5 mm along different axes were recorded. Prostate position and couch position were plotted before, during and after each couch shift. There was no visible impact of couch shifts on prostate motion. The standard deviation of prostate position was calculated before, during and after each couch shift. The standard deviation did not significantly increase during couch shifts (by 3 % on average, *p* = 0.88) and even slightly decreased after a couch shift (by 37 % on average; *p* = 0.02).

**Conclusions:**

Shifts of the patient couch did not adversely affect the motion of the prostate relative to the patient couch. Hence, shifts of the patient couch may be a viable way to correct the position of the prostate relative to the dose distribution.

## Findings

### Background

In external beam radiotherapy, tumor control probability and normal tissue toxicity [1–5] are strongly correlated to the ability to deposit dose within the limits of the clinical target volume. In case of the prostate, three-dimensional ultrasound is quite a precise image guidance modality both for patient positioning before each fraction [6–9] and for monitoring of the intra-fraction motion of the prostate [10–12].

As the mismatch between target volume and dose coverage accumulates due to intra-fraction motion, one way to remove the residual error is to perform a couch shift in opposite direction. Ideally, the motion of the table (relative to the beam) translates into the exact same motion of the prostate (relative to the beam). However, in principle such couch shifts, due to accelerating forces, could cause secondary displacements of the patient and prostate (relative to the couch) or even aggravate intra-fraction motion. The aim of this study is to confirm or reject this possibility.

## Patients and methods

Fifteen patients with histologically confirmed adenocarcinoma of the prostate were included in this analysis. All patients received norm-fractionated IMRT with 6 MV photons in our institution with a cumulative dose ranging from 70.0 to 76.0 Gy, depending on the tumor stage. Average age of patients was 72.0 ± 9.0 years (median 76.4 years, range 53.2 to 85.9 years).

**Fig. 1:**
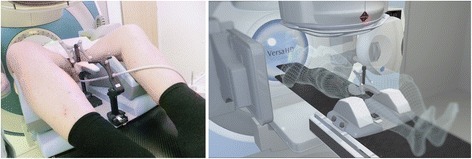
The auto-scan probe of the Clarity system has contact with the perineum of a patient (*left image*, photo credit Minglun Li). The illustration by the manufacturer (*right image*, image credit Elekta AB, Stockholm, Sweden) shows the integration of the ultrasound probe (*center*) into the treatment environment with gantry (*top*), cone beam CT (*left and right*) and robotic couch (*bottom*)

Intra-fraction motion of the prostate was tracked during 359 fractions by four-dimensional perineal ultrasound. The Clarity ultrasound system (see Fig. 1) was used in combination with an auto-scanning perineal ultrasound probe [10] which provided estimates of the prostate position at a rate of about one to two Hertz. During this time, a total of 22 couch shifts of up to 31.5 mm along different axes were recorded.

For each couch shift, the (absolute) position of the couch and the position of the prostate (relative to the couch) were plotted for 3 min, centered on the shift. The plots were evaluated for any visible impact of couch movements on prostate motion relative to the couch.

Prostate motility was defined as the standard deviation of prostate positions. It was calculated for each 1 min before, during, and after each couch shift.

## Results

**Fig. 2:**
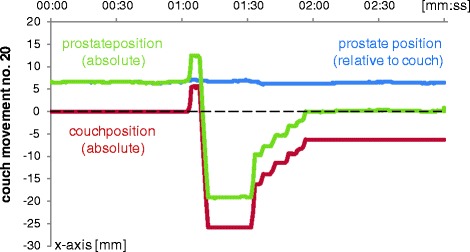
The prostate follows the couch shifts. A net shift of the couch of 6.5 mm corrects the prostate position to zero. The couch moves at a speed of 9.7 mm per second. The amplitude of the couch shift amounts to 31.5 mm. Still, neither the speed nor the magnitude of the couch shift affects the intra-fraction motion of the prostate (prostate position relative to the couch). The amplitude of intra-fraction motion is less than 1 mm, and the correlation coefficient between relative prostate position and couch position is only −0.06

**Fig. 3:**
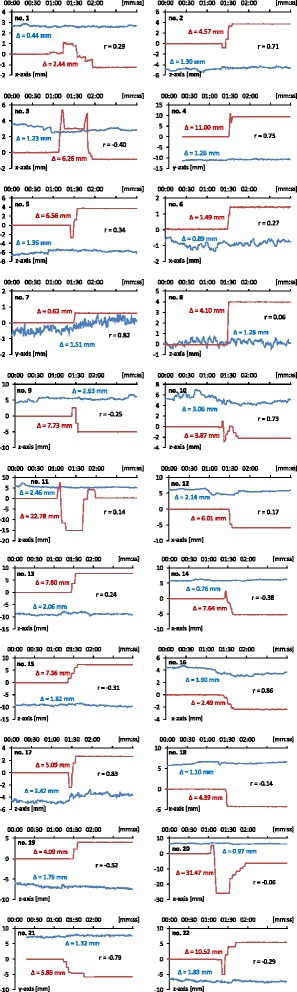
Intra-fraction motion of the prostate (*blue plots*) was not visibly affected by couch shifts (*red plots*) during any of the 22 recorded couch movements. In most cases, couch shift amplitudes (red Δ) were several times larger than prostate motion amplitudes (blue Δ). In particular, amplitudes of intra-fraction motion did not increase during couch movements. The correlation coefficient (r) between prostate motion and table shifts was mostly insignificant

The prostate position closely followed along the couch position in every case. Consider the example in Fig. 2. It was selected because of all recordings it features the largest amplitude of couch shifts. At 00:00, the prostate was off by 6.5 mm in absolute terms (relative to the beam isocenter), and the couch position was at zero (relative to the beam isocenter). Between 01:00 and 02:00 several couch shifts were performed, with a net offset of the couch of −6.5 mm (relative to the beam isocenter). The prostate closely followed all of these shifts (relative to the beam isocenter) such that there was no remaining residual error after the shifts, with the prostate at 0.0 mm after 02:00. During all of these shifts, the position of the prostate relative to the table continued its intra-fraction motion, however at much smaller amplitude and not visibly affected by even the largest shifts at a speed of 9.7 mm per second. Similar patterns of motion were observed during the 22 recorded couch shifts, all of which were plotted in Fig. 3. In no case there was any visible influence of the couch shifts (relative to the beam isocenter, red lines in Fig. 3) on intra-fraction motion of the prostate (relative to the couch, blue lines in Fig. 3).

**Fig. 4:**
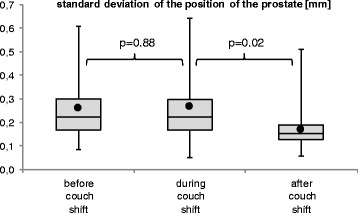
Intra-fraction motion of the prostate did not increase during couch shifts and even somewhat decreased after. The amount of intra-fraction motion is here shown as the standard deviation of prostate position. It was calculated for each 1 min before, centered around, and after couch shifts. It’s average value of 0.26 mm before couch shifts did not significantly increase during couch shifts to 0.27 mm (+3 %, *p* = 0.88). After couch shifts, motility seemed to somewhat decrease, in fact

The standard deviation of prostate positions (relative to the couch) as a measure of prostate motility was calculated for each of the 3 min shown in the plots. Average motility before the shifts was 0.26 mm ± 0.14 mm (mean ± SD, median 0.22 mm, range 0.08 to 0.61 mm); average motility during the shifts was 0.27 mm ± 0.16 mm (mean ± SD, median 0.22 mm, range 0.05 to 0.64 mm); average motility after the shifts was 0.17 mm ± 0.09 mm (mean ± SD, median 0.15 mm, range 0.06 to 0.51 mm); see Fig. 4.

Thus, motility did not significantly increase during couch shifts (by 3 % on average, *p* = 0.88) and even slightly decreased after a couch shift (by 37 % on average; *p* = 0.02). The decrease in motility after a couch shift could be a statistical artifact. Tentatively, it could be because patients tautened when experiencing couch shifts. In any case, this effect needs further measurements for confirmation and explanation.

## Conclusions

Couch shifts for real time correction of intra-fractional setup errors did not result in any relevant secondary motion of the prostate. In particular, motility did not significantly increase during couch shifts.

In conclusion, the strategy of real time correction of intra-fractional setup errors seems feasible as far as our results with regard to possible secondary motion of the prostate are concerned.

## Consent

Written informed consent was obtained from the patient for the publication of the accompanying image.
